# Phosphodiesterase-III Inhibitor Prevents Hemorrhagic Transformation Induced by Focal Cerebral Ischemia in Mice Treated with tPA

**DOI:** 10.1371/journal.pone.0015178

**Published:** 2010-12-06

**Authors:** Mitsunori Ishiguro, Keisuke Mishiro, Yasuyuki Fujiwara, Huayue Chen, Hiroshi Izuta, Kazuhiro Tsuruma, Masamitsu Shimazawa, Shinichi Yoshimura, Masahiko Satoh, Toru Iwama, Hideaki Hara

**Affiliations:** 1 Molecular Pharmacology, Department of Biofunctional Evaluation, Gifu Pharmaceutical University, Gifu, Japan; 2 Departments of Neurosurgery, Gifu University Graduate School of Medicine, Gifu, Japan; 3 Laboratory of Pharmaceutical Health Sciences, School of Pharmacy, Aichi Gakuin University, Aichi, Japan; 4 Departments of Anatomy, Gifu University Graduate School of Medicine, Gifu, Japan; Julius-Maximilians-Universität Würzburg, Germany

## Abstract

The purpose of the present study was to investigate whether cilostazol, a phosphodiesterase-III inhibitor and antiplatelet drug, would prevent tPA-associated hemorrhagic transformation. Mice subjected to 6-h middle cerebral artery occlusion were treated with delayed tPA alone at 6 h, with combined tPA plus cilostazol at 6 h, or with vehicle at 6 h. We used multiple imaging (electron microscopy, spectroscopy), histological and neurobehavioral measures to assess the effects of the treatment at 18 h and 7 days after the reperfusion. To further investigate the mechanism of cilostazol to beneficial effect, we also performed an in vitro study with tPA and a phosphodiesterase-III inhibitor in human brain microvascular endothelial cells, pericytes, and astrocytes. Combination therapy with tPA plus cilostazol prevented development of hemorrhagic transformation, reduced brain edema, prevented endothelial injury via reduction MMP-9 activity, and prevented the blood-brain barrier opening by inhibiting decreased claudin-5 expression. These changes significantly reduced the morbidity and mortality at 18 h and 7 days after the reperfusion. Also, the administration of both drugs prevented injury to brain human endothelial cells and human brain pericytes. The present study indicates that a phosphodiesterase-III inhibitor prevents the hemorrhagic transformation induced by focal cerebral ischemia in mice treated with tPA.

## Introduction

Accumulating evidence suggests that, for acute ischemic brain attack, it is a fact that thrombolysis is beneficial for patients with an ischemic stroke if given during the first 4.5 h of symptoms (NINDS, ECASS III) [1], [2]. Beyond this time window, delayed tissue plasminogen activator (tPA) does not appear to be as beneficial and actually increases the risk of serious side effects. Conditionally, delayed thrombolysis may be related to increased risk of brain edema and hemorrhagic transformation and other potential side effects [3]. Therefore, any way to reduce tPA-associated blood-brain barrier (BBB) injury may extend the time window for safe and effective reperfusion therapy, and ultimately increase the overall efficacy of tPA thrombolytic therapy.

Cilostazol, a selective inhibitor of phosphodiesterase III, is an antiplatelet drug and a vasodilator via an increased cyclic AMP (cAMP) level and cyclic GMP level [4], [5]. Cilostazol has been approved and used as a vasodilating antiplatelet drug for the treatment of ischemic symptoms in chronic peripheral arterial obstruction or intermittent claudication and for secondary prevention of cerebral infarction (CSPS I) [6]. Cilostazol has also been used after aneurismal SAH to prevent development of delayed cerebral vasospasm [7]. Recently, cilostazol has been shown to be a more effective and safer alternative to aspirin for long-term prevention of the recurrence of ischemic stroke in patients with chronic ischemic stroke [8].

On the other hand, researchers have reported that cilostazol has a neuroprotective effect against ischemic brain injury and prevents attenuated acute brain infarction induced by middle cerebral artery (MCA) occlusion (MCAO) and reperfusion in rats [9]-[10], which suggests that cilostazol has the potential to ameliorate acute ischemic stroke by minimizing evolving ischemic injury. We further reported that cilostazol protects against hemorrhagic transformation in mice transient focal cerebral ischemia [11] and combination treatment with normobaric hyperoxia, cilostazol protects mice against focal cerebral ischemia [12], and cilostazol provided neuroprotection against filamental MCAO-mediated increases in metallothionein-1 and -2 [13]. In addition, increasing evidence indicates that cilostazol may offer endothelial protection via both an inhibition of lipopolysaccharide-induced apoptosis and an inhibition of neutrophil adhesion to endothelial cells [14]. Since endothelium is one of the main constituents of BBB, cilostazol may provide not only endothelial protection but also BBB protection. The above suggests that cilostazol may not only be neuroprotective, but also protect against hemorrhagic transformation induced by tPA during reperfusion after ischemia. By so doing, cilostazol may extend the therapeutic time window for thrombolytic treatment. Very recently, cilostazol has been reported to be more effective than aspirin in the secondary prevention of all types of stroke in patients and, in particular, prevent the secondary attack of hemorrhagic stroke in patients (CSPS II) [15]. Indeed, a characteristic feature of cilostazol is that it has weaker hemorrhagic side effects than other antiplatelet drugs and does not increase the bleeding time [16]. Accordingly, the aim of this study was to assess whether tPA-induced hemorrhagic transformation is indeed suppressed by acute cilostazol treatment in an MCAO model and observe an electron microscopic view of microvessels to elucidate the mechanism underlying tPA-induced hemorrhagic transformation.

## Results

### Cerebral Infarction and Hemorrhagic Transformation Induced by tPA

**Figure 1:**
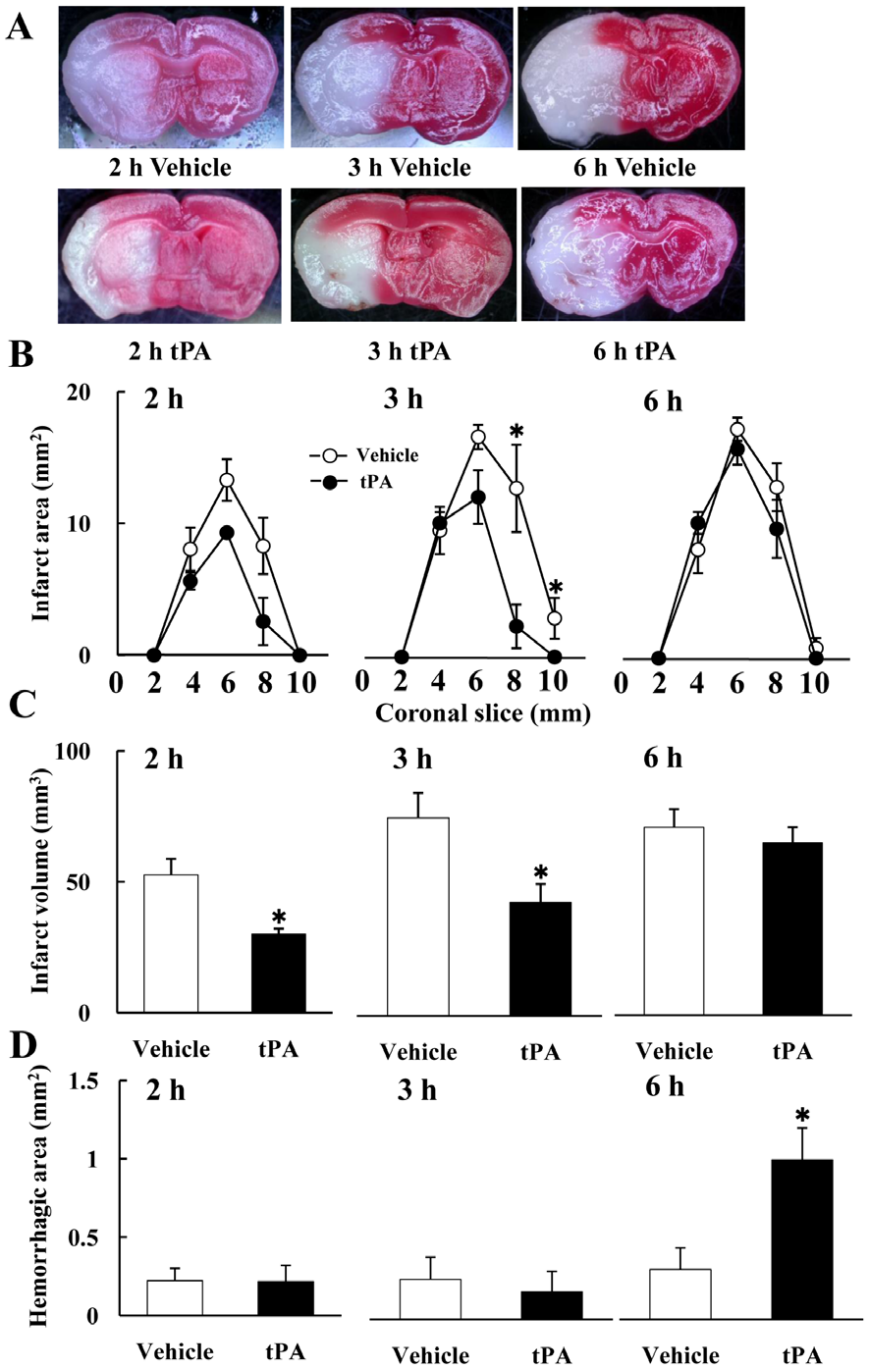
Effects of tPA on cerebral infarction and hemorrhage at 18 h after reperfusion. TTC-stained coronal sections show infarct tissues (pale unstained region). Brain sections (from extreme left) were acquired from 2 h vehicle, 3 h vehicle, 6 h vehicle, respectively. They were acquired from 2 h tPA (left), 3 h tPA, and 6 h tPA, respectively (A). These figures show the effects of tPA on the cerebral infarct area (B), volume (C), and hemorrhage area (D). tPA at 2 h and 3 h significantly decreased the infarct area and volume. Delayed tPA at 6 h had no effect on infarction but induced cerebral hemorrhage. *P<0.05 vs. vehicle (Welch's *t* test, n = 5–8). Data are expressed as means ± SEM.

**Figure 2:**
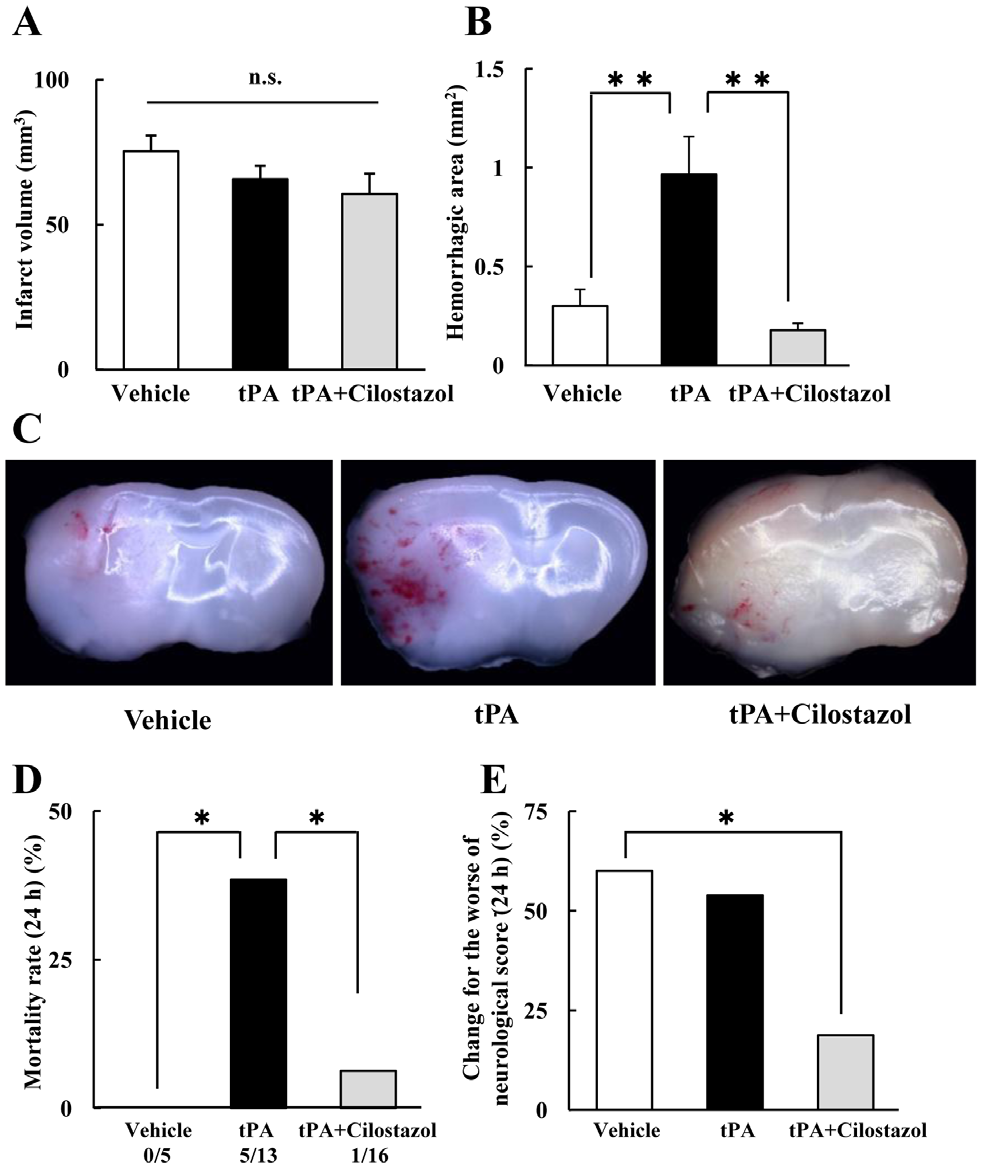
Effects of tPA and cilostazol on cerebral infarction and hemorrhage at 18 h after reperfusion. The other figures show the effects of tPA and cilostazol regarding the cerebral infarct volume (A) and hemorrhage area (B). Combination therapy with tPA plus cilostazol at 6 h had no effect on infarction but prevented hemorrhage. *P<0.01 vs. tPA (Welch's *t* test, n = 8–11). Coronal sections of brains (from extreme left) were acquired from vehicle, tPA, tPA plus cilostazol with no staining (location; 1 mm anterior to bregma) showing hemorrhagic spots in the infarct area (C). 34 animals were treated. Mortality rates were 0% (0/5) in saline-treated mice and 38% (5/13) in the delayed tPA group, whereas combination therapy with tPA plus cilostazol treatments significantly reduced mortality to 6.25% (1/16) at 18 h after reperfusion (D). *P<0.05 vs. tPA (χ^2^ test, n = 5–16). Furthermore, combination therapy significantly prevented neurological deficits (E). *P<0.05 vs. vehicle (χ^2^ test, n = 5–16). Data are expressed as mean ± SEM.

Early tPA treatment at 2 or 3 h significantly reduced infarct areas and volumes at 24 h after MCAO, but no efficacy was obtained with the delayed 6-h tPA administration (Fig. 1A–C). Instead, delayed tPA treatments induced detectable amounts of hemorrhagic transformation in the ischemic brain (Fig. 1D). Combination therapy with 6-h tPA plus cilostazol did not improve the infarct areas (data not shown) or volumes (Fig. 2A) after delayed tPA at 6 h, but improved the hemorrhagic transformation (Fig. 2B and 2C). No mice had died at 24 h after 2 h and 3 h MCAO in the vehicle and tPA groups.

### Neurological Function Induced by tPA

The ischemic mice showed apparent neurological deficits just after and at 18 h after reperfusion.

There was no significant difference in the neurological deficit score among the three groups just after reperfusion. In contrast, at 18 h after reperfusion, the combination group exhibited a significantly reduced mortality rate and prevented worsening of the neurological deficit scores (Fig. 2D and 2E).

### Acute BBB Injury, Intracerebral Hemorrhage, Brain Edema, and MMP-9 Activity Induced by tPA

**Figure 3:**
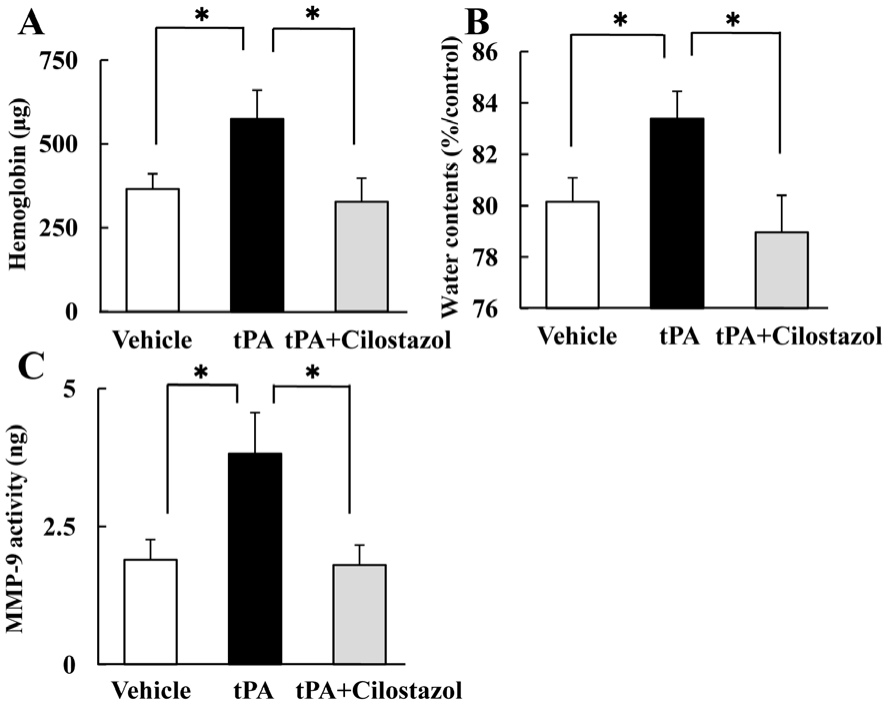
Effects of tPA and cilostazol on acute brain injuries at 18 h after reperfusion. These figures show the effects of tPA and cilostazol regarding hemorrhage volume (A), blood-brain barrier permeability (B), and MMP-9 expression (C) measured. Combination therapy with tPA plus cilostazol significantly prevented this tPA-associated amplification of cerebral hemorrhage, water content, and MMP-9 activity. *P<0.05 vs. tPA (Welch's *t* test, n = 7–10). Data are expressed as means ± SEM.

tPA administered at the delayed 6 h induced significantly the hemorrhagic transformation at 18 h after reperfusion. The combination of tPA plus cilostazol at 6 h significantly ameliorated the severity of hemorrhagic transformation (Fig. 3A). Correspondingly, combination therapy significantly prevented tPA-induced brain edema at 18 h after reperfusion (Fig. 3B). Our results demonstrate that cilostazol effectively inhibited tPA-induced amplification of cerebral hemorrhage and brain edema. The expression of MMP-9 was remarkably upregulated by tPA (Fig. 3C). The MMP-9 activity was significantly decreased with tPA plus cilostazol at 6 h. The activity of MMP-9 paralleled the degree of the hemorrhage volume in the ischemic brain. These findings suggest that cilostazol suppressed tPA-induced intracerebral hemorrhage by reducing the activity of MMP-9.

### Subacute Brain Injury Induced by tPA

**Figure 4:**
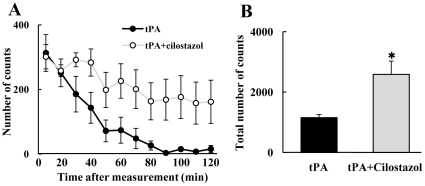
Effects of tPA and cilostazol on subacute brain injuries at 7 days after MCAO. These figures show the effects of tPA and cilostazol on neurobehavioral assessment (A and B) measured at 7 days after MCAO. Combination therapy with tPA plus cilostazol increased the total locomotor activity in the first 120 min of measurement. *P<0.05 vs. tPA (Student's *t* test, n = 5–7). Data are expressed as means ± SEM.

tPA alone significantly decreased mortality at 7 days after MCAO, but combination therapy with tPA plus cilostazol did not improve the mortality more than tPA alone (χ^2^ test, vehicle; n = 5/5, tPA; n = 8/13, tPA plus cilostazol; n = 9/16). To assess the effect of cilostazol on subacute brain injury, we used locomotor activity at 7 days after MCAO. In comparison with the tPA alone group, the tPA plus cilostazol group showed a significantly lower total activity count over the first 120 min (Fig. 4A and 4B).

### Electron Microscopic Analysis in Endothelial Cells

**Figure 5:**
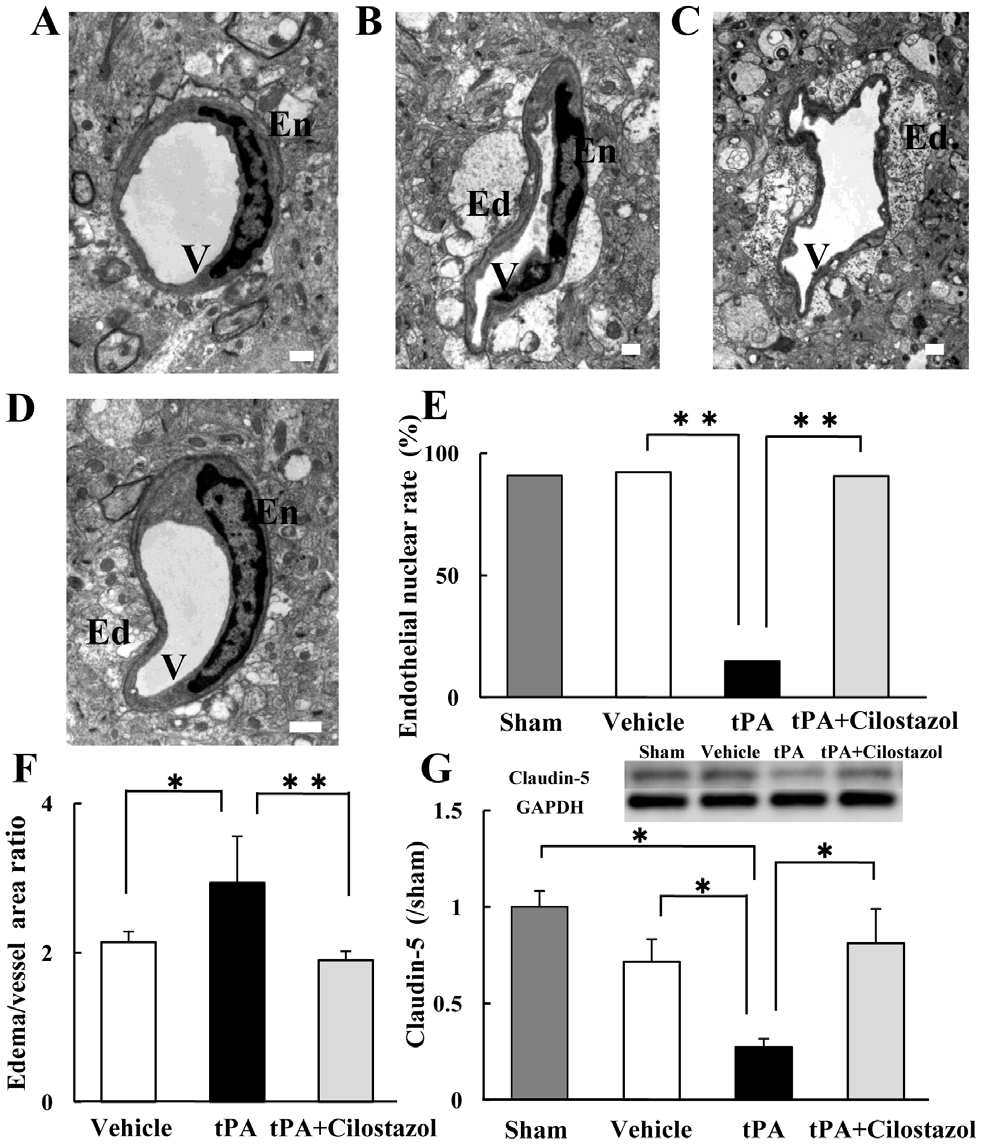
Effects of tPA and cilostazol on endothelial cells at 18 h after reperfusion. Electron microscopic view of microvessels in a peri-infarct area obtained from the sham model (A), the ipsilateral side of the vehicle model (B), tPA (C), and combination therapy with tPA plus cilostazol (D) Although no significant differences were observed in the nuclei of the endothelial cells of the sham and vehicle models at 6 h, the nuclei of the cells with tPA at 6 h significantly had disappeared. On the other hand, combination therapy with tPA plus cilostazol at 6 h significantly prevented the exacerbation induced by tPA (E). **P<0.01 vs. tPA (χ^2^ test, n = 11–32). Combination therapy with tPA plus cilostazol significantly decreased this tPA-associated brain edema (F). *P<0.05 vs. tPA (Student's *t* test, n = 5–10). Western blot analysis measured the levels of claudin-5. tPA alone at 6 h decreased claudin-5 levels. Combination therapy with tPA plus cilostazol prevented this tPA-associated decrease in claudin-5 (G). *P<0.05 vs. tPA (Welch's *t* test, n = 3–4). Data are expressed as means ± SEM. Ed; edema, En; endothelial cell, V; vessel. Scale bar = 1 µm.

An electron microscopic examination revealed that the nuclei of the endothelial cells had disappeared on the ipsilateral side in the brain tissue treated with tPA alone, though there were nuclei on the ipsilateral side in the brain tissue treated with vehicle. Furthermore, delayed tPA at 6 h significantly increased brain edema around the capillary arteries in the ischemic lesion. In contrast, combination therapy with tPA plus cilostazol significantly prevented the disappearance of the nuclei of the endothelial cells and reduced brain edema induced by tPA (Fig. 5A–F). These observations indicate that the mechanism of the neurovascular unit dissociated by tPA involved the injury of endothelial cells, which was prevented by cilostazol.

### Evaluation Claudin-5 Expression with Western Blotting Analysis

To characterize changes in the tight junction, we also evaluated the expression of claudin-5 with Western blotting. Interestingly, delayed tPA at 6 h markedly diminished claudin-5 levels in brain tissue. In contrast, combination therapy with tPA plus cilostazol at 6 h significantly prevented a decrease in claudin-5 expression (Fig. 5G).

### tPA and Cilostazol in Various Cell Cultures

**Figure 6:**
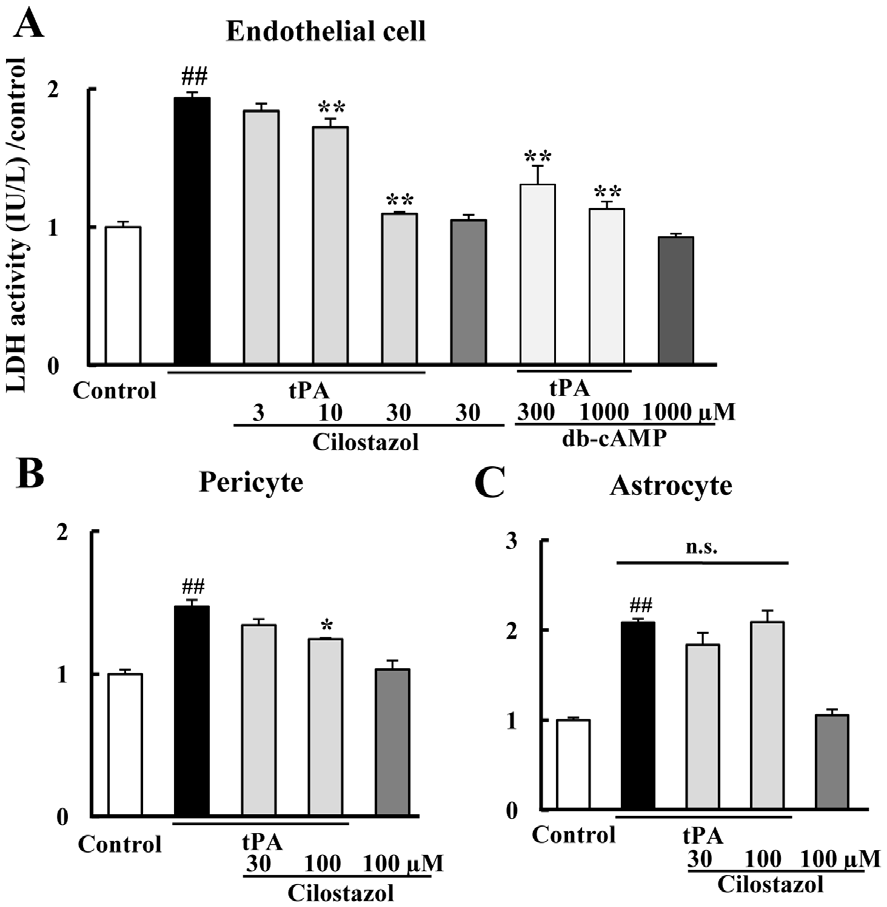
Effects of cilostazol on the tPA-stimulated human brain microvascular endothelial cells, pericytes, and astrocytes. Cell damage after 300-µg/ml tPA treatment was assessed by measuring LDH release into the culture medium in endothelial cells (A), pericytes (B), and astrocytes (C). The LDH level was increased by 300-µg/ml tPA treatment, and the increase was reduced by cilostazol at 30 and 100 µM in endothelial cells, and by cilostazol at 100 µM in pericytes. In addition, db-cAMP at 300 and 1000 µM significantly prevented cell damage induced by tPA in endothelial cells. ^##^P<0.05 vs. no treatment, *P<0.05, **p<0.01 vs. 300-µg/ml tPA alone (Dunnett's test, n = 4–8). Data are expressed as means ± SEM.

To assess the effects of combination treatment with tPA and cilostazol on the neurovascular unit, we evaluated the neuronal damage in endothelial cells, pericytes, and astrocytes. In endothelial cells, the LDH level was not changed by 3, 10, 30, and 100 µg/ml of tPA (data not shown) but was increased by 300-µg/ml tPA, and the increase was significantly reduced by the combination of 10 to 100 µM of cilostazol in a concentration-dependent manner (Fig. 6A). On the other hand, in pericytes and astrocytes, the LDH level was not changed by 3, 10, 30, and 100 µg/ml of tPA (data not shown) but increased by 300-µg/ml tPA. Furthermore, the increase was significantly reduced by the combination of 100 µM cilostazol in pericytes but not in astrocytes (Fig. 6B, C). We next examined the effect of db-cAMP on cell damage in tPA-treated endothelial cells. db-cAMP at 300 and 1000 µM significantly prevented the cell damage induced by tPA in a concentration-dependent manner (Fig. 6A). Given the average plasma concentration of cilostazol orally administered to humans (100 mg/body/day) is about 2 to 10 µM and may be partially higher in our body, the concentration of cilostazol we used is comparable to a clinical concentration [17], [18].

## Discussion

We have previously reported that cilostazol monotherapy reduced the infarct volume, hemorrhagic area, and brain edema in mice subjected to MCA occlusion and reperfusion [11]. In the present study, we examined whether cilostazol prevented the hemorrhagic transformation induced by tPA and found that cilostazol reduced the extent of hemoglobin content, water content, and MMP-9 activity in mice subjected to MCA occlusion and reperfusion, supporting the idea that cilostazol limits or prevents BBB disruption after the ischemic injury.

The tPA-induced breakdown of the barrier permeability of BBB often accelerates the progression of diseases such as cerebral ischemia. However, the molecular mechanisms involved with the barrier disruption by tPA have not been determined completely. Identification of the molecules responsible for the tPA-induced disruption of endothelial barrier may yield new therapeutic targets of intractable diseases. Therefore, we focused on claudin-5, which is a member of the claudin family and is involved in the assembly of tight junctions between microvascular endothelial cells [19], [20]. Our electron microscopic view revealed that the endothelial cells were severely degraded, the nuclei of the cells had disappeared, and the inner lumen had become thin in the tPA-treated mice. In the previous report, tPA increased apoptotic vessels compared to the vehicle treated mice after ischemia-reperfusion [21]. An induction of the apoptotic cascade seems to be involved in rtPA cytotoxicity on human corneal endothelial cells [22]. Therefore, the disappearance of the nuclei of the endothelial cells with tPA treatment may be due to apoptosis. With this in vivo model, the degree of claudin-5 was reduced in the mouse ischemic brain treated with tPA. Cilostazol prevented a decrease in claudin-5 expression. These changes were essentially identical to those observed in electron microscopic views of the mice treated with tPA and tPA plus cilostazol. Our results showed that the expression of claudin-5 was altered by tPA, which results in the disruption of the barrier permeability of neural vascular endothelial cells, and whose disruption was prevented by cilostazol. Previous study suggested that the cause of BBB disruption is because of a proteolytic degradation of the vascular membrane in cerebral ischemia [23]. Among proteases, matrix metalloproteinases (MMP), in particular MMP-2 and MMP-9, are able to digest the endothelial basal lamina and therefore may play a pivotal role in promoting BBB permeability [24]. Accumulating evidence suggests that MMP-9 activation is closely related to those side effects of t-PA [25]. Jin et al. have reported that loss of claudin-5 signals appears to be ameliorated in CD47 knockout mice and the genetic deletion of CD47 reduces MMP-9 expression [26]. Taken together, in the present study, cilostazol could be used to upregulate claudin-5 and downregulate the overall inflammatory MMP-9 responses.

The concept of the “neurovascular unit” has emerged as a new paradigm for understanding the pathology of CNS diseases, including stroke, in recent years [27]. In order to truly preserve brain tissue and function, one may have to conserve all the complex signals and interactions between a network of multiple cell types, including neurons, astrocytes, and microvascular endothelial cells. Therefore, we examined the effects of cilostazol on tPA-induced cell damage in human brain microvascular endothelial cells, pericytes, and astrocytes. tPA at a concentration of 300 µg/ml induced cell damage in all three types of cell cultures; cilostazol at 10 and 30 µM protected microvascular endothelial cells and cilostazol at 100 µM protected pericytes, but cilostazol at 30 or 100 µM did not protect astrocytes against cell damage induced by tPA. Ballabh at al. have reported that endothelial cells are directly responsible for the formation of barriers, while astrocytes and pericytes regulate the barrier function of endothelial cells. In addition, compared to endothelial cells in other tissues, the BBB endothelial cells have more extensive tight junctions [28]. As described above, the three types of cells that comprise the BBB are different in various points. However, a growing body of evidence indicates that tPA mediates a cerebral ischemia-induced increase in the permeability of the neurovascular unit. From these points of view, cilostazol may protect against cell damage induced by tPA in endothelial cells and pericytes comprising the neurovascular unit, presumably resulting in improvement in the neurologic score and the survival rate.

Cilostazol increases intracellular cAMP content accordingly and activates protein kinase A (PKA) and PI3K/Akt signaling [29]. cAMP analogs enhance the barrier function of tight junctions in brain capillary endothelial cells [30]. cAMP-dependent induction of claudin-5 expression could be involved in the promotion of tight-junction function in endothelial cells [31]. Furthermore, phosphorylation of Akt may be involved in determining cell survival or cell death after transient focal cerebral ischemia [32]. Cilostazol induces NO production by eNOS activation via a cAMP/PKA- and PI3K/Akt-dependent mechanism [29]. From these points of view, we next evaluated whether cilostazol protects the cell damage by cAMP activation. The present study demonstrated that db-cAMP prevented the cell damage induced by tPA in human brain microvascular endothelial cells (Fig. 6A), and this result may correlate with the result that cAMP activity may be, in part, the mechanism under combination therapy that protects against neuronal damage induced by tPA and prevents hemorrhagic transformation with functional and morphological outcomes.

The present findings indicate that cilostazol may be one of the potential drugs to reduce hemorrhagic complications induced by tPA. However, there are several limitations in the present study. One limitation may be that we only examined infarct and hemorrhagic volumes of the combination treatment at 18 h after stroke. Namely, it remains to be rigorously determined whether the acute neurovascular protection sustains for longer periods in tissue recovery, and how it interfaces with tPA effects in delayed times post-stroke. Another limitation may be whether the effect of acute cilostazol treatment on hemorrhagic transformation induced by MCAO was consistent with that of combination therapy of tPA and cilostazol. Further studies may be warranted to dissect the underlying cell signaling mechanisms of combination therapy, to determine the optimum dosage, to test whether combined cilostazol therapy could extend the short therapeutic window of tPA, and to validate the possibility of a combined therapy for future clinical application.

In conclusion, cilostazol protected against not only acute but also subacute tPA-induced cerebral injury in the mouse that had undergone a hemorrhagic transformation, prevented the loss of claudin-5, and inhibited tPA-induced cell damage in human brain microvascular endothelial cells. These effects may be due to the protection of endothelial cells, at least in part, the neurovascular unit in association with cAMP activity. The present findings indicate that a phosphodiesterase-III inhibitor (cilostazol) may be a potential drug for reducing hemorrhagic complications induced by tPA.

## Materials and Methods

### Animal Preparation

The experimental designs and all procedures were approved by the Animal Experiment Committee of Gifu Pharmaceutical University (permission number; 09-176, 222, 282, 308, 362, and 402). All procedures relating to animal care and treatment conformed to animal care guidelines of this committee. All in vivo experiments were performed using male ddY mice (4 weeks old; body weight 22–28 g; Japan SLC Ltd., Shizuoka, Japan).

### Experimental Groups and Drug Treatments

This study consisted of two different experiments. Experiment 1 aimed to investigate the evaluation of the therapeutic time window of tPA (Grtpa, Mitsubishi Tanabe Pharma Corporation, Osaka, Japan) at 2 h, 3 h, and 6 h after MCAO. Mice were randomly assigned to two groups (vehicle; n = 6–8, tPA; n = 5–7). Experiment 2 aimed to investigate the evaluation that cilostazol (Pletaal, Otsuka Pharmaceutical Corporation, Tokushima, Japan) prevents hemorrhagic transformation-associated damage induced by tPA at 6 h after MCAO. Mice were randomly assigned to three groups (vehicle; n = 10, tPA; n = 9, tPA plus cilostazol; n = 8). A dose of 10 mg/kg of tPA in 0.1 ml saline was administered intravenously just before a reperfusion [34]. A dose of 10 mg/kg of cilostazol in 0.5% carboxymethyl cellulose was injected intraperitoneally followed by tPA. The injection volume was adjusted to 8 ml/kg body weight. Mice assigned to the vehicle group received an intravenous injection of 0.1 ml saline without tPA at a timing and volume similar to that used in the tPA group. The experiments performed blinded for treatment group.

### Focal Cerebral Ischemia

To imitate the clinical situation, we designed the present experiment whereby tPA was administrated just before a reperfusion by pulling out a nylon monofilament from the origin of MCA, so that the tPA-associated damage could be assessed under similar conditions of reperfusion [33]. Mice were anesthetized with 2.0 to 2.5% isofluorane in 30% oxygen and 70% nitrous oxide via a facemask (Soft Lander; Sin-ei Industry, Saitama, Japan). An 8-0 nylon monofilament (Ethicon, Somerville, NJ, USA) via the internal carotid artery, as described by Hara et al [34], was used to induce focal cerebral ischemia. After occlusion for 2, 3 and 6 h, which could cause hemorrhagic infarction after tPA treatment [35], the nylon was gently withdrawn to restore the blood flow in the MCA territory and the incision was closed. In all animals during surgery, the body temperature was maintained between 37.0 and 37.5°C with the aid of a heating lamp and heating pad. To confirm MCAO, laser-Doppler flowmetry (Omegaflow flo-N1; Omegawave Inc., Tokyo, Japan) measured the regional artery blood flow (rCBF) in the MCA territory from the temporal bone surface [12]. Mice that did not demonstrate a significant reduction just before reperfusion (to less than 40% that of the contralateral rCBF values) were excluded.

### Tissue Processing

At 18 h after reperfusion, mice were given an overdose of pentobarbital sodium (Dainippon Sumitomo Pharma, Osaka, Japan) and transcardially perfused with cold saline. The mice brains were removed immediately, and the brain was cut into 5 serial 2-mm-thick coronal block slices. In our preliminary study, we found that, among all slices, the reproducibly largest infarct size was in the cortical region of the left hemisphere on the anterior 4-th slice. Therefore, we used this area to analyze the extent of intracerebral hemorrhage, matrix metalloproteinase (MMP) -9 activity, and claudin-5 [36].

### Measurement of Infarct Volume and Hemorrhagic Area

At 18 h after reperfusion, these slices were immersed for 20 min in a 2% solution of 2, 3, 5-triphenyltetrazolium chloride (TTC) (Sigma-Aldrich, St Louis, Mo, USA). Image J measured the unstained areas of the total infarctions, and the infarction volume was calculated as in a previous report [37]. These areas of the total hemorrhagic spots as scarlet dot lesions within the area of ischemic damage were measured using Image J, and the hemorrhagic area was calculated.

### Neurological Deficits

Mice (vehicle; n = 5, tPA; n = 13, tPA plus cilostazol; n = 16) were tested for neurological deficits at 24 h or 7 days after MCAO. These were scored as described in our previous study [34].0, no observable neurological deficits (normal); 1, failure to extend the right forepaw (mild); 2, circling to the contralateral side (moderate); 3, loss of walking or righting reflex (severe); 4, dead. The investigator who rated the mice was masked as to the group to which each mouse belonged.

### Spectrophotometric Assay of Hemoglobin

Hemorrhagic transformation was quantified with a spectrophotometric assay of brain hemoglobin content [38]. After adding 10 ml/g saline to individual samples, they were homogenized and followed by 30 min centrifugation (13,000 rpm). Then 200 µl reagent (QuantiChrom Hemoglobin Assay Kit; BioAssay Systems, Hayward, Calif, USA) was added to 50 µl supernatant. After 15 min, optical density was measured at 400 nm with a spectrophotometer (Skan It RE for Varioskan 133 Flash 2.4; Thermo Fisher Scientific,Waltham, MA, USA). Total hemoglobin content was expressed as micrograms per samples (vehicle; n = 9, tPA; n = 10, tPA plus cilostazol; n = 7).

### Measurement of Brain Water Content

At 18 h after reperfusion, the brains were divided between the edematous area and the non-edematous area, each of which was weighed to obtain the wet weight and then dried at 110°C for 24 h. The water content in the edematous area was calculated as water content (%) = (wet ·weight – dry ·weight)/wet ·weight ×100 [39] (vehicle; n = 9, tPA; n = 10, tPA plus cilostazol; n = 7).

### Assay of MMP-9 Activity

To measure MMP-9 activity, each sample was adjusted to the same protein content. We followed the instructions of the manufacturer of the MMP-9 Biotrak activity assay system kits (R&D Systems, Minneapolis, Minn, USA) to assess MMP-9 activity in brain extracts. The total MMP-9 activity was expressed as nanograms per samples. (vehicle; n = 8, tPA; n = 10, tPA plus cilostazol; n = 7).

### Measurement of Locomotor Activity

Mice were individually placed in a transparent acrylic cage (175×245×125 mm) with sawdust bedding on the floor, and digital counters with infrared sensors (NSASS01; Neuroscience, Inc., Tokyo, Japan) measured locomotion every 10 min for 120 min. This test was performed once per animal on day 7. [12] (tPA; n = 5, tPA plus cilostazol; n = 7).

### Electron Microscopy

At 18 h after reperfusion, mice were anesthetized with pentobarbital sodium and transcardially perfused with cold saline solution followed by 2.5% glutaraldehyde in 0.1M phosphate buffer, PH 7.4. After perfusion, the brains were removed and then dissected. As control group, sham-operated mice were used. These sections were cut on a Porter-Blum MT-1 ultra microtome (DuPont-Sorvall, Wilmington Del, USA), stained with uranyl acetate and lead salts, and examined with a JEOL 1010 transmission electron microscope (JEOL, Tokyo, Japan). Moreover, three levels of sections were selected as described above, and five areas in the ipsilateral peri-infarct cortex in each section were chosen. In addition, we counted the nuclei of endothelial cells larger than 1 µm (sham; n = 13, vehicle; n = 13, tPA; n = 29, tPA plus cilostazol; n = 41), chose some vessels and measured the area of each brain swelling and blood vessels of the capillary artery using Image J. We calculated the ratio of the blood vessel/brain swelling (vehicle; n = 10, tPA; n = 5, tPA plus cilostazol; n = 8). Sham-operated mice were subjected to the same surgical procedure, except that the nylon monofilament was not advanced to occlude the MCA.

### Western Blot Analysis

As control group, sham-operated mice were used. Tissues were homogenized in 10 ml/g tissue ice-cold lysis buffer (50 mM Tris-HCl, pH 8.0, containing 150 mM NaCl, 50 mM EDTA, 1% Triton X-100, and protease/phosphatase inhibitor mixture). A sample of 5 µg of protein was subjected to a 15% gradient SDS-polyacrylamide gel electrophoresis (SuperSep Ace; Wako Pure Chemicals, Osaka, Japan), with separated protein being transferred onto a polyvinylidene difluoride membrane (Immobilon-P; Millipore Corporation, Billerica, MA, USA). For immunoblotting, the following primary antibodies were used: polyclonal antibody to claudin-5 (1∶500 dilution; Zymed) and monoclonal antibody to GAPDH (1∶5000 dilution; Cell Signaling). The secondary antibody was anti-rabbit HRP-conjugated IgG (1∶5000 dilution). The SuperSignal West Femto Maximum Sensitivity Substrate (Thermo Fisher Scientific, Waltham, Mass, USA) was used to visualize the immunoreactive bands. A Lumino Imaging Analyzer (FAS-1000; Toyobo Engineering, Osaka, Japan) and a Gel Pro Analyzer (Media- Cybernetics, Inc., Bethesda, MD) were used to measure the band indensity (sham; n = 3, vehicle; n = 4, tPA; n = 3, tPA plus cilostazol; n = 3).

### Cell Cultures

Human brain microvascular endothelial cells (DS Pharma Biomedical, Osaka, Japan) were cultured in 100-mm dishes at 37°C in HuMedia EG2 (Kurabo, Osaka, Japan) supplemented with 2% heat-inactivated fetal bovine serum under a humidified 5% CO_2_ atmosphere until they reached confluency. They were then transferred into 24-well culture plates and cultured until they became confluent. Human brain pericytes and astrocytes (DS Pharma Biomedical) were transferred into 24-well culture plates and cultured in CS-C medium (DS Pharma Biomedical) supplemented with 10% heat-inactivated fetal bovine serum until they became confluent. The medium was discarded, and the cells were washed twice. Then, we exposed the cells to tPA alone (10, 30, 100, and 300 µg/ml), tPA (300 µg/ml) plus cilostazol (3, 10, 30, and 100 µM) for 24 h. In addition, we exposed endothelial cells to tPA (300 µg/ml) plus cilostazol (30 µM) plus db-cAMP (dibutyryladenosine 3′5′ -cyclic monophosphate sodium salt; Sigma-Aldrich, Madison, Wis, USA) (300 and 1000 µM) for 24 h. After the cells were treated, the conditioned medium was harvested and used to determine the LDH kit (Promega, Madison, Wis, USA), a marker of nonspecific cell damage.

### Statistical Analysis

Data are presented as means ± SEM. Statistical comparisons were made using an analysis of variance followed by a Student's *t* test, Welch's *t* test, Dunnett's test, or χ^2^ test. *P*<0.05 was considered to indicate statistical significance.
